# Differences in Emotion Regulation Considering Gender, Age, and Gambling Preferences in a Sample of Gambling Disorder Patients

**DOI:** 10.3389/fpsyt.2019.00625

**Published:** 2019-09-11

**Authors:** Marta Sancho, Marta de Gracia, Roser Granero, Sara González-Simarro, Isabel Sánchez, Fernando Fernández-Aranda, Joan Trujols, Núria Mallorquí-Bagué, Gemma Mestre-Bach, Amparo del Pino-Gutiérrez, Teresa Mena-Moreno, Cristina Vintró-Alcaraz, Trevor Steward, Neus Aymamí, Mónica Gómez-Peña, José Manuel Menchón, Susana Jiménez-Murcia

**Affiliations:** ^1^Department of Psychiatry, Hospital de la Santa Creu i Sant Pau, Barcelona, Spain; ^2^Department of Psychiatry, Bellvitge University Hospital-IDIBELL, L’Hospitalet de Llobregat, Spain; ^3^CIBER Fisiopatología Obesidad y Nutrición (CIBERobn), Instituto de Salud Carlos III, Madrid, Spain; ^4^Department of Psychobiology and Methodology, Autonomous University of Barcelona, Barcelona, Spain; ^5^Department of Clinical Sciences, School of Medicine, University of Barcelona, Barcelona, Spain; ^6^CIBER Salud Mental (CIBERsam), Instituto de Salud Carlos III, Madrid, Spain; ^7^Nursing Department of Mental Health, Public Health, Maternal and Child Health, Nursing School of the University of Barcelona, Barcelona, Spain

**Keywords:** behavioral addiction, emotion regulation, gambling disorder, risk, severity, age, gender

## Abstract

**Introduction:** Impairments in emotion regulation are understood to be a transdiagnostic risk factor of suffering from compulsive and addictive behaviors. The aim of this study was to investigate the role of emotion regulation deficits in gambling disorder and to analyze these differences taking gender, age, and gambling activity preferences into account. Methods: The sample included n = 484 patients seeking treatment for gambling disorder at a specialized outpatient service. Main outcomes were sociodemographic variables, emotion regulation, and gambling severity. Results: Differences between sexes were found in non-acceptance of emotions. Older patients obtained higher levels in non-acceptance of emotions, lack of emotion regulation strategies, emotional clarity, and global emotion regulation scores. No differences were found in emotion scores considering gambling preferences (non-strategic versus strategic). Path analysis showed that emotion regulation scores and age had a direct effect on gambling disorder severity, while emotion regulation and gambling preference were not mediational variables in the relationships of gender and age with gambling severity. Conclusions: Emotion regulation impairments differ in patients seeking treatment for gambling problems. Early prevention and intervention programs should incorporate the different dimensions of this process, taking into account clinical phenotypes.

## Introduction

Impairments in emotion regulation (ER) are understood to be a transdiagnostic risk factor for the onset and maintenance of addictions (1–5). According to Gross (6, p. 275), ER refers to “those processes for which individuals exert an influence on their emotions, when have them and how experience and express them.” Other models such as that by Gratz and Roemer (7) focus on difficulties in ER, underlying that healthy ER should consist in the capacity to modulate rather than suppress negative emotional states (8). These authors name several components of *emotional dysregulation*, such as impaired awareness of emotions or an inability to refrain from impulsive behavior when experiencing negative emotions. Studies focusing on clinical samples have shown that individuals with ER deficits often engage in maladaptive behaviors to downregulate or to escape from their emotions (1, 5, 9). These difficulties in managing emotions can lead to a lack of coping skills and bring about errors in self-regulation and impulse control, which constitute a risk factor for several disorders such as behavioral addictions (5, 10, 11).

Concerning gambling disorder (GD; 12), although different subgroups can be identified, with differentiated clinical, psychopathological, and personality characteristics, ER problems are usually present in most people affected by this disorder (13–15). Rogier and Velotti (8) conceptualize this addiction in a model that integrates ER components described in Gratz and Roemer’s model and the timeline model by Sheppes et al. (16) of healthy ER processing. In this way, these authors refer to difficulties in ER processes among the individuals with GD. For instance, pathological gamblers show deficits in identifying emotions because of a failure in emotional awareness, a difficulty in accepting emotional states, and poor ER self-efficacy. In line with these results, Williams et al. (1) found a lack of emotional clarity and emotional awareness as well as a high impulsivity in a sample with GD. Other authors reported greater difficulties in describing feelings as well as higher scores in externally oriented thinking in this population compared to healthy controls (17–19). Orlowski et al. (20) associated a lack of reappraisal or an accepting attitude toward negative emotions with the number of *Diagnostic and Statistical Manual of Mental Disorders, 5th Edition* (*DSM-5*) symptoms, indicating a greater GD severity. Consistent with previous studies, GD is defined as an affect regulation disorder in which the gambling serves as a coping strategy (21). These findings emphasize the role that emotion dysregulation and other related constructs, such as alexithymia, play in the vulnerability to GD (9).

A potentially addictive behavior like gambling could be used as a coping strategy in the presence of negative emotional states (22, 23), especially in the case of *emotionally vulnerable problem gamblers* (13). Along these lines, GD research has described different groups of gamblers depending on the nature of ER motivations. For instance, McCormick and Taber (24) described two types of gamblers based on arousal levels: over-stimulated or under-stimulated gamblers. This last type is similar to gamblers with alexithymia, described by Bonnaire et al. (25, 26), who are characterized by a need for continuous stimulation, participating in exciting activities such as strategic gambling (e.g., sport bets or poker) (27). In addition, these individuals present greater GD severity than those with a preference for non-strategic gambling (e.g., slot machines or bingo). This passive gambling (non-strategic gambling) is understood as an avoidance strategy when faced with depressive symptoms. As a whole, this suggests that gambling preference/s seem to be clinically significant and provide a way of subtyping individuals with gambling-related problems (27–29).

Other variables, such as gender or age, have a core role in the relationship between ER and GD. Studies carried out in the general population showed significant differences in ER profiles related to age and gender. Positive emotional experiences and the use of strategies to maintain them are more frequent with age (30, 31), whereas more negative and changing emotional states are experienced during adolescence (32–34). In this regard, older people refer to a greater ability in using ER strategies (35, 36). However, ER competence decreases among older people with high levels of emotion activation, for example, in those who have experienced negative life events (35, 36). In relation to gender, a greater tendency toward rumination in women could account for increased anxiety and affective disorders (37, 38). It has also been suggested that women’s higher scores in distraction could lead them to use gambling more frequently as a way to escape or to avoid stressful situations and daily life frustrations (39–41).

Previous research has mainly focused on gambling preferences and GD profiles. However, no study to date has addressed underlying mechanisms (including direct and indirect effects) between ER and other variables, such as gender and age. ER impairments can have a significant impact on the treatment outcome (42, 43). For this reason, identifying the variables associated with ER difficulties is crucial for designing treatments that are more specific and tailored to each patient, with the aim of increasing the response to treatment (43). Therefore, those patients with greater ER difficulties could benefit from specific treatments such as mindfulness (42, 43) or serious games aimed at improving these capabilities (44).

Based on the theoretical framework mentioned above, the aims of the current study were: a) to evaluate differences in ER domains based on gender, age, and gambling preferences; b) to examine the predictive capacity of ER domains on GD severity and to explore the potential moderating role of gender, age, and gambling subtype; and c) to conduct a path analysis model valuing the underlying mechanism between gender, age, gambling preference, ER, and gambling severity. We hypothesized that female gender, strategic gambling, and young age would predict ER deficits, and that these difficulties would be associated with greater gambling severity.

## Methods

### Participants

The sample consisted of 484 patients (450 men and 34 women) with gambling-related problems, consecutively recruited and receiving outpatient treatment at the Gambling Disorder Unit at the Department of Psychiatry of Bellvitge University Hospital between 2015 and 2017. All patients were over 18 years of age. Exclusion criteria were the presence of an organic medical illness or neurodegenerative condition (such as Parkinson’s disease); a psychotic disorder; and current (or history of) brain injury, a neurological disease, or intellectual disabilities.

### Measures

*Diagnostic Questionnaire for Pathological Gambling* according to *DSM* criteria (45). This scale includes 19 items to assess *DSM* diagnostic criteria for GD, based on both the *DSM, Fourth Edition, Text Revision* (*DSM-IV-TR*) and *DSM-5*. The Spanish adaptation of the questionnaire has good psychometric properties (α = 0.81 for general population and α = 0.77 for GD clinical sample; 46). This study analyzed the total number of *DSM-5* criteria for GD as a measure of gambling behavior severity.

*Difficulties in Emotion Regulation Scale (DERS;* 7*).* This self-report questionnaire contains 36 items that assess difficulties in regulating emotions. It is composed of six first-order scales: 1) non-acceptance of emotional responses, 2) difficulties engaging in goal-directed behavior, 3) impulse-control difficulties, 4) lack of emotional awareness, 5) limited access to effective ER strategies, and 6) lack of emotional clarity. A total scale is also available as a global measure of emotion dysregulation. This study used the Spanish version of the questionnaire, which has demonstrated adequate psychometric features (47).

*Sociodemographic and Clinical Variables.* Patients completed a semi-structured face-to-face interview regarding GD, psychopathological symptoms, and personality traits (48). The same interview also collected sociodemographic data (e.g., education, occupation, marital status) and additional clinical information, such as gambling preferences (non-strategic versus strategic gambling). Social status was measured using the Hollingshead index, a survey based on educational level and occupational prestige (49).

**Table 1** includes the internal consistency (Cronbach’s alpha, α) for the psychometric scales used in the study. Effect sizes for these coefficients, according to Cicchetti (50), were in the fair range (α = 0.714 for DERS lack of emotional awareness scale) to excellent (α = 0.930 for DERS total scale).

**Table 1:** Sample description (n = 484).

Sociodemographic variables		*n*	*%*
Gender	*Female*	34	7.0%
	*Male*	450	93.0%
Origin	*Spain*	447	92.4%
	*Other country*	37	7.6%
Marital status	*Single*	221	45.7%
	*Married/partner*	192	39.7%
	*Separated/divorced*	71	14.7%
Education level	*Primary*	273	56.4%
	*Secondary*	177	36.6%
	*University*	34	7.0%
Social index	*Medium-high to high*	38	7.8%
	*Medium*	48	9.9%
	*Medium-low*	190	39.3%
	*Low*	208	43.0%
Employment	*Unemployed*	171	35.3%
	*Employed*	313	64.7%
Clinical variables	*α*	*Mean*	*SD*
Age (years)		41.27	13.21
GD onset (years)		29.07	11.80
GD duration (years)		5.83	5.86
Mean bets—episode (euros)		167	542
Maximum bets—episode (euros)		2,000	6,561
Cumulate debts, present (euros)		9,681	20,745
*DSM-5* total criteria	.821	6.99	2.00
DERS non-acceptance of emotions	.886	17.38	6.73
DERS goal-directed behaviors	.797	14.11	4.82
DERS impulse control	.840	14.03	5.71
DERS lack of emotional awareness	.714	16.63	4.52
DERS emotion regulation	.891	19.56	7.84
DERS emotional clarity	.755	11.94	4.18
DERS total score	.930	93.52	24.87

SD, standard deviation; α, Cronbach’s alpha in the current study.

### Procedure

The assessment was conducted prospectively upon arrival to the treatment unit and prior to treatment during a face-to-face interview (with a mean duration of 90 min), in which the tests mentioned above were administered.

In this study, the differentiation between strategic versus non-strategic gambling was based on gambling activity, which represented the highest impairment for the participants and was reported as the main reason for seeking treatment.

### Statistical Analysis

Statistical analysis was carried out with Stata15 for Windows. Comparisons for mean scores on DERS scales were based on analysis of variance (ANOVA) procedures. Gender comparisons (women versus men) were adjusted for the covariates age and GD severity (*DSM-5* total criteria). Group age comparisons (two groups were compared based on the age median in the sample) were adjusted for patients’ gender and GD severity. Comparisons for gambling preference (non-strategic vs. strategic) were adjusted for patients’ gender, age, and GD severity. The effect size for mean differences was estimated through Cohen’s *d* coefficient (|*d*| > 0.20 was considered poor effect size, |*d*| > 0.5 was moderate, and |*d*| > 0.8 was large; 51). In this study, Finner’s procedure was used to control type I error due to multiple comparisons (this method is included in the family-wise error rate stepwise procedures and offers a more powerful test than Bonferroni correction; 52).

The predictive capacity of ER dimensions (DERS scores, defined as the independent variable) on GD severity (*DSM-5* total criteria for GD, defined as the criterion) was assessed with a lineal multiple regression. Independent models were obtained considering DERS first-order scales and DERS total scales, and the modeling was run in three blocks: a) the first block included and set the participants’ gender, age, and gambling preference; b) the second block added DERS scale scores; and c) the third block added and tested the interactions between each DERS scale and gender, age, and gambling subtype. The final model only retained those significant interaction parameters (*p* ≤ 05), obtained main effects for DERS scales that had no significant interaction parameters and single effects for DERS scales with significant interactions. The predictive capacity of the final model was measured through the adjusted R^2^ coefficient, and the predictive capacity of each block through the change in the adjusted R^2^.

Structural equation modeling (SEM) was conducted to test the underlying mechanisms between the patients’ gender, age, gambling preference (non-strategic vs. strategic), ER levels (DERS scale scores), and GD severity (*DSM-5* total criteria). Specifically, path analysis was run using the maximum likelihood (ML) estimator, and the overall goodness-of-fit was evaluated through standard statistical measures (53): the root mean square error of approximation (RMSEA), Bentler’s comparative fit index (CFI), the Tucker–Lewis index (TLI), and the standardized root mean square residual (SRMR). RMSEA < 0.10, TLI > 0.9, CFI > 0.9, and SRMR < 0.1 were considered adequate model fit.

## Results

### Sample Characteristics

**Table 1** includes the description of the sociodemographic and clinical variables of the study sample. Most participants were men (93%), born in Spain (92.4%), single (45.7%) or married (or living with a stable partner, 39.7%), with low education levels (56.4% completed primary schools), employed (64.7%), and with middle-low (39.3%) to low (43.0%) social position index. The mean age was 41.3 years (SD = 13.2), the mean age of GD onset was 29.1 years (SD = 11.8), and the mean duration of GD was 5.8 years (SD = 5.9). Regarding gambling variables, the mean number of *DSM-5* criteria for GD was 7 (SD = 2), and 56.8% of the patients reported debts related to gambling behaviors. Based on the GD severity classification of *DSM-5*, n = 31 patients were in the problematic group (6.4% of the participants did not meet clinical criteria for GD diagnosis and reported between one and three symptoms), n = 66 in the low-severity group (13.6%, with four or five symptoms), n = 158 in the moderate-severity group (32.6%, with six or seven symptoms), and n = 229 in the severe group (47.3%, with eight or nine symptoms).

### Associations Between Emotion Regulation and Gender, Age, and Gambling Preference

**Table 2** contains the results of the ANOVA procedures (adjusted for age and GD severity) in the study [all the models fulfilled goodness-of-fit, with non-significant results (*p* > .05) in the lack-of-fit tests]. The upper part of this table includes the comparison of mean DERS scores for both genders (ANOVA adjusted for age and GD severity). Significant differences were only found for the non-acceptance of emotions scale: men showed higher mean scores than women (17.6 versus 15.1, *p* = .031).

**Table 2:** Comparison of the DERS scores based on gender, age, and gambling preference.

	Women *(n = 34)*		Men *(n = 450)*		ANOVA adjusted for patients’ age and gambling severity			
	*Mean*	*SD*	*Mean*	*SD*	*MD*	*F-stat*	*p*	|*d*|
DERS non-acceptance of emotions	15.13	5.17	17.55	6.82	2.42	4.655	.031*	0.40
DERS goal-directed behaviors	13.93	4.54	14.12	4.85	0.19	.058	.810	0.04
DERS impulse control	14.50	5.57	13.99	5.72	0.50	.287	.593	0.09
DERS lack of emotional awareness	16.82	3.78	16.62	4.57	0.20	.058	.810	0.05
DERS emotion regulation	18.85	6.37	19.62	7.94	0.77	.343	.558	0.11
DERS emotional clarity	11.99	4.49	11.94	4.16	0.05	.005	.942	0.01
DERS total score	91.20	20.69	93.70	25.18	2.50	.380	.538	0.11
	^1^Age: 18–40 *(n* = *252)*		^1^Age: 41–75 *(n* = *232)*		ANOVA adjusted for gender and gambling severity			
	*Mean*	*SD*	*Mean*	*SD*	*MD*	*F-stat*	*p*	|*d*|
DERS non-acceptance of emotions	16.19	6.61	18.68	6.80	2.48	18.251	<.001*	0.37
DERS goal-directed behaviors	13.85	4.86	14.39	4.79	0.53	1.623	.203	0.11
DERS impulse control	13.75	5.90	14.33	5.50	0.58	1.434	.232	0.10
DERS lack of emotional awareness	16.41	4.38	16.87	4.67	0.47	1.200	.274	0.10
DERS emotion regulation	18.28	7.79	20.97	7.85	2.69	15.754	<.001*	0.34
DERS emotional clarity	11.56	4.10	12.37	4.27	0.81	4.642	.032*	0.19
DERS total score	89.83	25.16	97.61	24.50	7.77	13.611	<.001*	0.31
	Non-strategic *(n* = *346)*		Strategic *(n = 138)*		ANOVA adjusted for gender, age, and gambling severity			
	*Mean*	*SD*	*Mean*	*SD*	*MD*	*F-stat*	*p*	|*d*|
DERS non-acceptance of wemotions	17.63	17.72	16.76	16.54	0.88	1.667	.197	0.05
DERS goal-directed behaviors	13.87	13.80	14.71	14.88	0.84	2.968	.086	0.06
DERS impulse control	13.89	13.82	14.37	14.56	0.48	.698	.404	0.03
DERS lack of emotional awareness	16.57	16.66	16.79	16.57	0.22	.201	.654	0.01
DERS emotion regulation	19.61	19.65	19.46	19.35	0.15	.036	.849	0.01
DERS emotional clarity	11.95	11.97	11.93	11.88	0.03	.004	.952	0.00
DERS total score	93.26	93.38	94.14	93.87	0.88	.130	.719	0.01

*^1^Groups of age defined by the median of the chronological age in the sample (40 years old).*

*SD, standard deviation; MD, mean difference.*

*Bold: significant comparison (.05).

The middle section of **Table 2** contains the mean comparisons between the two age groups (classification that was based on the median—percentile 50—age of the sample, 40 years old), adjusted for gender and GD severity. Higher means corresponded to participants in the older age group for DERS non-acceptance of emotions, limited access to effective ER strategies, lack of emotional clarity, and total scales.

The lower part of **Table 2** includes the comparison of DERS subscale scores based on gambling preferences (non-strategic versus strategic). No statistically significant differences between both groups were found.

### Predictive Model

**Table 3** includes the two models valuing the predictive capacity of ER dimensions on GD severity, as well as the potential moderating role of gender, age, and gambling preference. The block assessing the interaction parameters between DERS scores and participants’ gender, age and, gambling preference was not significant (block/step 3 of model 1: *F* = 1.18, *df* = 18/440, *p* = .272; block/step 3 of model 2: *F* = 1.27, *df* = 3/460, *p* = .285), indicating that the contribution of ER to gambling severity is not dependent on/moderated by these features. Considering the regression including DERS first-order scales, gambling severity was higher for younger patients and those with greater levels in ER scales measuring non-acceptance of emotions and impulse control. Regarding the regression modeling for DERS total scale (model 2), gambling severity was also higher for younger participants and those with higher DERS total scores.

**Table 3:** Predictive capacity of emotion regulation on gambling severity (DSM-5 total criteria).

Model 1: DERS first-order scales	*B*	*SE*	*Beta*	*T*	*p*	*95% CI for B*	
Gender (0 = women/1 = men)	−0.184	0.322	−0.024	−0.570	.569	−0.817	0.450
Age (years)	−0.031	0.007	−0.202	−4.459	<.001*	−0.044	−0.017
Gambling type (0 = non-strategic/1 = strategic)	0.096	0.195	0.022	0.493	.622	−0.286	0.478
DERS: non-acceptance of emotions	0.040	0.019	0.135	2.147	.032*	0.003	0.077
DERS: goal-directed behaviors	0.044	0.026	0.106	1.692	.091	−0.007	0.094
DERS: impulse control	0.064	0.022	0.184	2.888	.004*	0.020	0.107
DERS: lack of emotional awareness	0.017	0.020	0.038	0.830	.407	−0.023	0.056
DERS: emotion regulation	0.018	0.020	0.071	0.912	.362	−0.021	0.057
DERS: emotional clarity	0.025	0.024	0.052	1.014	.311	−0.023	0.073
Model 2: DERS total score	*B*	*SE*	*Beta*	*T*	*p*	*95% CI for B*	
Gender (0 = women/1 = men)	−0.202	0.320	−0.026	−0.630	.529	−0.831	0.427
Age (years)	−0.032	0.007	−0.210	−4.709	<.001*	−0.045	−0.019
Gambling type (0 = non-strategic/1 = strategic)	0.117	0.192	0.027	0.611	.542	−0.260	0.495
DERS: total	0.036	0.003	0.453	11.179	<.001*	0.030	0.043

B, non-standardized parameter; SE, standard error; Beta, standardized parameter.

*Bold: significant parameter (.05 level).

### Pathways Analysis

**Figure 1:**
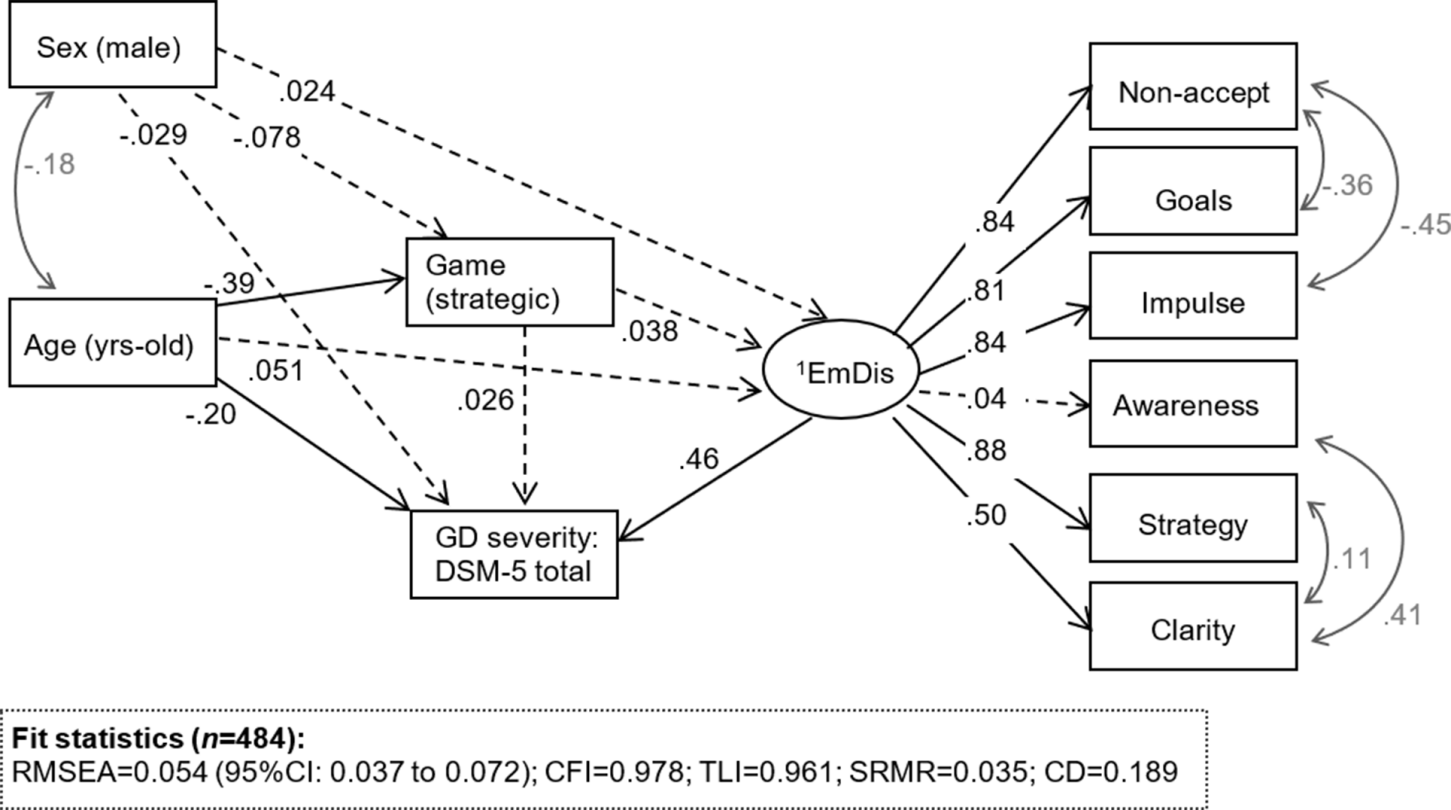
Sem model for the whole sample, standardized coefficients. Note. ^1^EmDis: emotional dysregulation (latent variable defined with the DERS first order scales). Continous line: significant coefficient (.05 level). Dash-line: non-significant coefficient. Grey: covariance parameter

**Figure 1** includes the path diagram with the standardized coefficients obtained in SEM (**Table S1**, **Supplementary Material**, contains complete model results). The independent variables of the model were gender (0 = female, 1 = male) and age (years). The dependent variable was GD severity (*DSM-5* total criteria), and the mediational variables were gambling preference (0 = non-strategic; 1 = strategic) and ER levels (a latent variable was defined as a global measure of ER difficulties based on the raw scores on the first-order factors of the DERS). Adequate goodness-of-fit was obtained: RMSEA = 0.054 (95% CI: 0.037 to 0.072), CFI = 0.978, TLI = 0.961, and SRMR = 0.035. The global predictive capacity for the model was around 19%.

Regarding the latent variable, all DERS subscales obtained significant and high loadings (higher than 0.30), except for the awareness dimension. DERS scores obtained a direct effect on GD severity (the higher the scores, the higher the GD severity), and age also registered a direct effect on this criterion (the younger the patients, the higher the number of GD *DSM-5* criteria). No direct effect of gender and gambling preference on gambling severity was found. DERS scores and gambling preference were not mediational variables between gender or age and gambling severity.

## Discussion

The present study analyzed the role of ER, as measured on the DERS, on GD severity and the potential differences in gender, age, and gambling preference. The main results of our study demonstrate higher levels of the non-acceptance of emotions among men, worse ER scores in older patients, and no significant differences in ED regarding gambling preferences (comparing non-strategic versus strategic activities). Path analysis showed direct effects of age and ER scores on GD severity and that ED and gambling preference did not mediate the relationship between gender or age and GD severity.

Contrary to hypothesized, the comparison of ER profiles by gender showed more difficulties in ER for men compared to women (specifically, men had poorer performance in the non-acceptance of emotions domain). A possible explanation of this finding could be due to the fact that men tend to adopt less adaptive strategies, namely, emotional suppression rather than cognitive reappraisal (54). On the contrary, women show greater predisposition to report their emotional states and to use more adaptive ER strategies than men (55–58). This greater emotional awareness has been observed in women even from an early age (59–61). Similarly, some studies suggest non-aware and automatic ER in men on leisure activities (62–64), showing a tendency to suppress emotional experience (35). This increased difficulty in accepting emotions could drive men to get involved in distracting activities as an experiential avoidance strategy and also lead to other forms of psychopathology (such as alcohol abuse) (39).

Also, contrary to our hypothesis regarding age, in this sample, older patients showed higher scores in three first-order scales of DERS (limited access to effective ER strategies, lack of emotional clarity, and non-acceptance of emotions) as well as in DERS total scores. Based on the available data, we would expect worse performance in ER for younger patients (32–34). One possible explanation accounting for our results could come from GD itself: patients’ involvement in persistent, uncontrollable, and unwanted gambling behavior generates significant emotional discomfort (distress), which could increase maladaptive ER strategies even at older ages. This hypothesis requires further empirical research, however. In line with this, some pioneering studies suggest that some strategies, such as emotional suppression, may be a useful form of ER for managing stressors in older age, which entails a possible decoupling of the psychological distress with age (65). However, these results must be evaluated with caution, since they have not assessed the specific contribution of ER domains in clinical samples of GD patients.

No significant differences between gambling preference (comparing non-strategic and strategic gambling) and ER profile in our sample were found. A possible explanation for our results could be the psychometric measures used. Perhaps the inclusion of other measures of ER and related constructs would yield different results. In line with this, studies have related gambling preference with alexithymia. For instance, Bonnaire et al. (26) demonstrated problems of alexithymia in individuals with a preference for strategic gambling (e.g., sport bets or poker) and depressive symptoms in those with a preference for non-strategic gambling (e.g., slot machines or bingo). Similarly, other authors (28, 29, 65) found a subtype of addicted gamblers who experience high levels of excitement and narcissistic traits. These individuals would be involved in strategic gambling, which induces elevated arousal though a high excitatory potential, suggesting possible alexithymia.

In the present study, gambling severity was higher for younger patients. So, GD severity was higher in patients with higher levels across several ER domains (e.g., non-acceptance of emotions and impulse control), as well as ER global measure. Greater GD severity in younger individuals could be explained by a preference of strategic gambling in this population. In addition, this subtype of gambling causes a higher level of gambling-related biases, such as illusion of control (66, 67), increasing the severity of the disorder. In line with this, previous studies showed that strategic gambling is associated with increased severity and with young gamblers, while non-strategic gamblers tend to be older and less severe (26, 68–71). Likewise, an early onset of GD is generally related to a severe presentation, as well as dysfunctional personality traits and greater psychopathology (46, 72). Other studies have shown less severity with advancing age, although medical problems and depression increase (68, 69). In accordance with other studies (25, 26, 73), in which ER-related constructs, such as alexithymia, were positively correlated with gambling severity, our results also indicated higher levels of ER deficits when gambling severity was higher. The severity of the disorder was higher in the younger group, but older patients showed greater levels of ER. Some studies have identified associations between psychopathology and older age (72), suggesting that gambling could be a dysfunctional strategy to cope with or to regulate negative emotions in these cases (74, 75).

Our study has several limitations that need to be considered when interpreting the results. Firstly, the sample of this work includes mainly men with medium-low to low social position levels and primary school education. Secondly, the number of women featured in the study is very small compared to men, which could decrease statistical power (that is, increase in the likelihood of a type II error and therefore the ability to detect the real effect of gender on the relationship between ER and gambling). It must be argued, however, that this study included all the patients consecutively arriving at the treatment unit, where the proportion of GD in men is higher. The number of women was enough to allow for statistical analyses. Thirdly, the size of the non-strategic gambler group was greater than the strategic gambling group, and this could have interfered in our results. Further studies with larger sample sizes are needed to increase statistical power and external generalization validity.

In spite of these limitations, the results of this study highlight the importance of taking into account gender, age, and gambling type during treatment. The severity presented by the younger patients and those with a high level of ER could hinder cognitive–behavioral therapy. Although this treatment is effective for GD, in these cases, other approaches could be considered (42, 76, 77). This subgroup could benefit from mindfulness-based interventions (MBI), such as mindfulness-based relapse prevention (MBRP; 78, 79). These types of treatment programs target the experiences of craving and negative affect. In addition, MBI is directly aimed at treating the experiential avoidance observed in these patients, improving their metacognitive capacities (80–83).

## Conclusions

This study provides greater understanding about the role of ER in GD. On one hand, our results suggest greater severity of GD among individuals with high ER deficits and in younger individuals. On the other hand, ER deficits are higher in men and older individuals. No differences in ER strategies based on gambling preference (strategic vs. non-strategic) were found. Future research should focus on exploring ER and other related constructs such as alexithymia, as well as on developing treatments that target difficulties in these aspects. Therefore, this study has been carried out through a statistical variable–centered approach, focused on the etiological objective of explaining the relationships and the underlying mechanisms between ER and gambling severity. Future research based on alternative and complementary statistical procedures such as person-centered approaches (e.g., mixture models or cluster analysis) should focus on identifying the dynamics of emergent therapeutic needs based on the categorizing of individuals into common subgroups based on substantive variables (such as ER domains) and on understanding the pattern of relationships of these empirical latent clusters with predictors, correlates, and therapy outcomes.

## Data Availability

All datasets generated for this study are included in the manuscript and the supplementary files.

## Ethics Statement

This study was carried out in accordance with the latest version of the Declaration of Helsinki. The Ethics Committee of the University Hospital of Bellvitge approved the study and written informed consent was obtained and signed from all final participants.

## Author Contributions

MS, SJ-M, IS, RG, and JMM were in charge of research project elaboration. MS, SJ-M, IS, and RG were in charge of organization. MS, SJ-M, IS, and RG were in charge of design. MS, MG, RG, NM-B, GM-B, AP-G, TM-M, CV-A, TS, NA, MG-P, and SJ-M were in charge of execution. Writing of the first draft was done by MS. Review and critique were done by MS, SJ-M, IS, JT, SG-S, and FF-A.

## Funding

This manuscript and research were supported by grants from the Instituto de Salud Carlos III (ISCIII) and cofounded by the FEDER funds/European Regional Development Fund (ERDF), a way to build Europe. CIBERobn and CIBERsam are initiatives of ISCIII. This work was also supported by the Ministerio de Economía y Competitividad (PSI2015-68701-R) and by the Delegación del Gobierno para el Plan Nacional sobre Drogas(2017I067).

## Conflict of Interest Statement

The authors declare that the research was conducted in the absence of any commercial or financial relationships that could be construed as a potential conflict of interest
