# Supplementary material for: Differences in Emotion Regulation Considering Gender, Age, and Gambling Preferences in a Sample of Gambling Disorder Patients
Source: Front Psychiatry. 2019 Sep 11;10:625. doi: 10.3389/fpsyt.2019.00625 (PMC6749049; doi:10.3389/fpsyt.2019.00625)
Supplement: Supplementary file 1 [file Table_1.docx]

Supplementary Material

*Table S1 (supplementary)*

*Results obtained in the SEM (standardized results)*

|  |  | Coeff. | SE | z | p | 95%CI coeff. | |
| --- | --- | --- | --- | --- | --- | --- | --- |
| DSM-5 | Gambling preference | 0.0256 | 0.0437 | 0.59 | 0.558 | -0.060 | 0.111 |
|  | Emotional dysregulation | 0.4641 | 0.0372 | 12.47 | <0.001 | 0.391 | 0.537 |
|  | Sex | -0.0286 | 0.0410 | -0.70 | 0.486 | -0.109 | 0.052 |
|  | Age | -0.2016 | 0.0439 | -4.59 | <0.001 | -0.288 | -0.116 |
| Gambling preference | Sex | -0.0777 | 0.0432 | -1.80 | 0.072 | -0.162 | 0.007 |
|  | Age | -0.3941 | 0.0399 | -9.87 | <0.001 | -0.472 | -0.316 |
| Emotional dysregulation | Gambling preference | 0.0382 | 0.0514 | 0.74 | 0.458 | -0.063 | 0.139 |
|  | Sex | 0.0239 | 0.0483 | 0.49 | 0.621 | -0.071 | 0.119 |
|  | Age | 0.0510 | 0.0522 | 0.98 | 0.328 | -0.051 | 0.153 |
| Emotional dysregulation | Non-acceptance | 0.8448 | 0.0232 | 36.34 | <0.001 | 0.799 | 0.890 |
|  | Goals | 0.8142 | 0.0200 | 40.62 | <0.001 | 0.775 | 0.853 |
|  | Impulse | 0.8358 | 0.0189 | 44.12 | <0.001 | 0.799 | 0.873 |
|  | Awareness | -0.0360 | 0.0478 | -0.75 | 0.452 | -0.130 | 0.058 |
|  | Strategy | 0.8751 | 0.0163 | 53.8 | <0.001 | 0.843 | 0.907 |
|  | Clarity | 0.5025 | 0.0368 | 13.66 | <0.001 | 0.430 | 0.575 |
| Covariance parameters | Non-acceptance, Goals | -0.3569 | 0.1088 | -3.28 | 0.001 | -0.570 | -0.144 |
|  | Non-acceptance, Impulse | -0.4527 | 0.1184 | -3.83 | <0.001 | -0.685 | -0.221 |
|  | Awareness, Clarity | 0.4102 | 0.0387 | 10.6 | <0.001 | 0.334 | 0.486 |
|  | Strategy, Clarity | 0.1139 | 0.0510 | 2.23 | 0.025 | 0.014 | 0.214 |
|  | Sex, Age | -0.1779 | 0.0448 | -3.97 | <0.001 | -0.266 | -0.090 |
